# A transfer RNA inosine modification drives genome-wide synonymous recoding across human commensal bacterial families

**DOI:** 10.1093/pnasnexus/pgag187

**Published:** 2026-05-30

**Authors:** Christopher D Katanski, Chathuri Pathirage, Jennifer C Chang, Iva Veseli, Tyler J Smith, Marcus Foo, Yuan Wu, Yichen Hou, Hoang Anh V Tran, Dominika Rudzka, Wen Zhang, Mohammad Amin Bayat Tork, Karen Lolans, Amy D Willis, Weixin Tang, A Murat Eren, Michael J Federle, Kristin S Koutmou, Tao Pan

**Affiliations:** Department of Biochemistry and Molecular Biology, University of Chicago, Chicago, IL 60637, USA; Department of Research and Development, MesoRNA LLC, Chicago, IL 60607, USA; Department of Chemistry, University of Michigan, Ann Arbor, MI 48109, USA; Center for Biomolecular Sciences, University of Illinois at Chicago, Chicago, IL 60607, USA; Helmholtz Institute for Functional Marine Biodiversity, Oldenburg, Lower Saxony 26129, Germany; Department of Chemistry, University of Michigan, Ann Arbor, MI 48109, USA; Committee on Microbiology, University of Chicago, Chicago, IL 60637, USA; Department of Chemistry, University of Chicago, Chicago, IL 60637, USA; Committee on Genomics, Genetics and Systems Biology, University of Chicago, Chicago, IL 60637, USA; Department of Biochemistry and Molecular Biology, University of Chicago, Chicago, IL 60637, USA; Department of Biochemistry and Molecular Biology, University of Chicago, Chicago, IL 60637, USA; Department of Biochemistry and Molecular Biology, University of Chicago, Chicago, IL 60637, USA; Department of Biochemistry and Molecular Biology, University of Chicago, Chicago, IL 60637, USA; Department of Medicine, University of Chicago, Chicago, IL 60637, USA; Department of Biostatistics, University of Washington, Seattle, WA 98195, USA; Department of Chemistry, University of Chicago, Chicago, IL 60637, USA; Helmholtz Institute for Functional Marine Biodiversity, Oldenburg, Lower Saxony 26129, Germany; Alfred Wegener Institute, Helmholtz Centre for Polar and Marine Research, Bremerhaven 27570, Germany; Institute for Chemistry and Biology of the Marine Environment, University of Oldenburg, Oldenburg, Lower Saxony 26129, Germany; Marine ’Omics Bridging Group, Max Planck Institute for Marine Microbiology, Bremen 28359, Germany; Center for Biomolecular Sciences, University of Illinois at Chicago, Chicago, IL 60607, USA; Department of Chemistry, University of Michigan, Ann Arbor, MI 48109, USA; Department of Biochemistry and Molecular Biology, University of Chicago, Chicago, IL 60637, USA; Committee on Microbiology, University of Chicago, Chicago, IL 60637, USA; Committee on Genomics, Genetics and Systems Biology, University of Chicago, Chicago, IL 60637, USA

**Keywords:** tRNA, inosine, codon evolution, genomics

## Abstract

Inosine modification on transfer RNA (tRNA) anticodon (I34) is universally conserved in three kingdoms of life and critical to tRNA decoding capabilities. We found that tRNA^Leu^(IAG) in commensal human bacterial families in Lactobaccilalles is concurrent with genome-wide synonymous leucine codon reprogramming. Pathway analysis reveals significant synonymous Leu codon changes in proteins in multiple KEGG pathways on cellular metabolism, where many genome-wide dominant UUA in families without tRNA^Leu^(IAG) is reprogrammed to CUU, CUC, and UUG in families with tRNA^Leu^(IAG). We provide biochemical and phenotypic results to support mechanisms that enable synonymous Leu codon substitutions to confer greater translation equivalency and growth fitness, indicating that a tRNA inosine modification can propel the genome-wide evolution of synonymous leucine codons.

Significance statement Katanski et al. investigates how transfer RNA (tRNA) modification propels the evolution of synonymous codons. Codon usage is highly selective in all organisms. Both mutations that change the amino acid identity and synonymous codon changes can be drivers of evolution. However, how codon evolution coordinates with tRNA modification remains unexplored. We show here that genome-wide leucine codons are drastically different among several human commensal bacterial families and co-occurs with an inosine modification in a leucyl-tRNA. Applying pathway analysis, leucyl-tRNAs in protein synthesis, and phenotypic studies of isogenic strains, we show that this tRNA I modification alters Leu decoding properties and confers an advantage in bacterial growth. A single tRNA modification can accommodate or even drive genome evolution.

## Introduction

Transfer RNAs (tRNAs) read the genetic code and are essential for translation. tRNAs have substantive sequence diversity between closely related microbial species and bear metabolically linked post-transcriptional modifications (1–3). Over 100 tRNA modifications have been reported across the three kingdoms of life (4). tRNA modifications are critical for tRNA structural stability and function, regulate tRNA folding dynamics, determine tRNA decoding fidelity, and fine-tune the tRNA-ribosome binding and translational kinetics (2, 5). Among them, tRNA anticodon modifications that occur at the wobble position (#34 in standard tRNA nomenclature) directly modulate decoding and contribute to decoding biases of synonymous codons.

tRNA modifications can act as an active component to engage in the evolution of coding sequences. One study (6) showed that the evolution of tRNA U34 or A34 modifications can explain the divergence of tRNA gene contents in bacterial and eukaryotic genomes. Codons read by the most abundant tRNA genes, when considering the anticodon modifications, are enriched in the genomes and positively correlate with translation efficiencies. Along the same lines, a genome-wide synonymous codon study of the *Drosophila* genus demonstrated a queuosine (Q34) modification conferring a selective advantage toward the NAC codons (7). Another genome-wide reprogramming is in *Candida albicans* where the appearance of a tRNA aminoacylated with serine but reading a standard Leu codon (CUG) leads to CUG recoding from Leu to Ser (8, 9). Another reprogramming occurs in plant pathogens from the *Streptomyces* genus where the coevolution of variants of prolyl-tRNA synthetase and tRNA^Pro^ leads to the translation of a standard Ala codon (GCU) as both Ala and Pro (10).

Inosine (I34) occurs through enzymatic deamination at A34 of tRNAs and is one of the few essential modifications that are highly conserved in bacteria and eukaryotes. The evolution of tRNA inosine modification follows the emergence of tRNAs with A34 in bacteria and eukaryotes, as the tolerance of the G:U wobble base pair by the ribosome eliminates the necessity of the simplest decoding strategies employed by archaea (11, 12). In bacteria, I34 is predominantly found on tRNA^Arg^(ACG), whereas in eukaryotes, I34 is present on eight different tRNAs. In this work, we study the emergence of I34 in tRNA^Leu^ in human commensal bacteria and its impact on translation, growth fitness, and the evolution of synonymous codons.

## Results and discussion

### tRNA^Leu^ anticodon modification co-occurs with synonymous codon disparity

**Figure 1:**
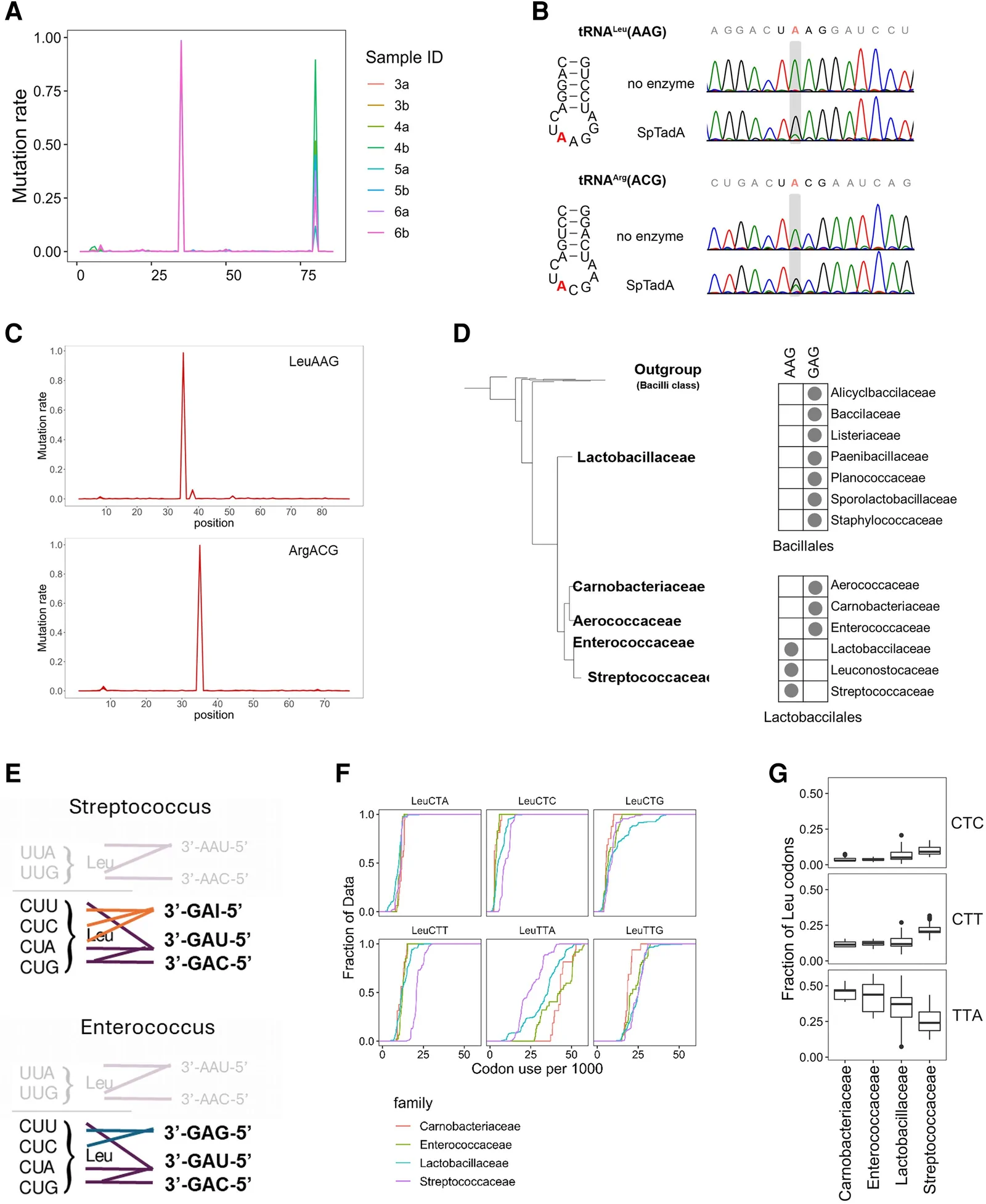
Bacterial tRNA^Leu^(AAG) gene coincides with genome-wide synonymous recording of leucine. A) tRNA-seq results from oral microbiome samples. Reads that map to tRNA^Leu^(AAG) from *Streptococcus* show a misincorporation signature at the wobble anticodon position 34, consistent with a tRNA modification from A to inosine. *n* = 8 biological replicates. B) Recombinant and purified TadA enzyme from *Streptococcus pneumoniae* was tested for deamination activity against in vitro transcribed tRNA^Leu^(AAG) and tRNA^Arg^(ACG). Sanger sequencing of the reverse transcribed reaction product shows A-to-G signal, consistent with A-to-I34 modification. C) tRNA-seq of a laboratory *Streptococcus mutans* culture showing A-to-I modification in tRNA^Leu^(AAG) and tRNA^Arg^(ACG) in the cultured cells. D) (Left) A phylogenomic tree of genomes in the Lactobacillales order, with outgroup genomes from the Alicyclobacillales, Bacillales, Paenibacillales, and Staphylococcales orders. Branches are truncated at the family level. (Right) Six bacterial families in the order Lactobacilales of class Bacilli consisting of three with a tRNA^Leu^(AAG) gene and three with a tRNA^Leu^(GAG) gene. No family contains both tRNA^Leu^(AAG) and tRNA^Leu^(GAG). All families in the order Bacillales of class Bacilli only contain the tRNA^Leu^(GAG) gene. E) The six synonymous Leucine codons are read by five tRNAs with overlapping decoding capabilities. F) Leu codon usage for species in four families in order Lactobacillales, two containing tRNA^Leu^(AAG) gene and two tRNA^Leu^(GAG) gene. Histograms show the distribution of codon usage among species in each family. *n* = 36, 72, 336, and 294 genomes for C, E, L, and S, respectively. G) Comparing RSCU among species in each of the four families in order Lactobacillales showing increased CUU and CUC usage along with decreased usage of UUA for families of Streptococcaceae and Lactobacillaceae containing a tRNA^Leu^(AAG) gene.

We applied tRNA sequencing to explore the bacterial metaepitranscriptome of the human oral cavity (Fig. S1A and B) (2, 13). During the course of surveying tRNA modifications in our data, we observed a significant misincorporation sequencing signature at position A34 in tRNA^Leu^(AAG) from Streptococcus family tRNAs (Fig. 1A). tRNAs with A at the wobble anticodon position (position 34 in standard tRNA nomenclature), are typically only studied for tRNA^Arg^(ACG) in bacteria, despite being widespread in multiple tRNA species in eukaryotes (6, 14). A34 in tRNA^Arg^(ACG) is enzymatically deaminated to inosine (I) in bacterial cells, an essential modification that expands the decoding capacity of tRNA^Arg^(ICG) to C/U/A-ending Arg codons (12, 15). The misincorporation signature in our sequencing data is consistent with A-to-I deamination and further supported by previously reported biochemical validation of tRNA^Leu^(IAG) from a closely related bacterium (11, 16). We validated the tRNA^Leu^(IAG) in Streptococcus species biochemically using in vitro deamination of the known Streptococcus tRNA^Arg^(ICG) deaminase, TadA (12) (Fig. 1B), and tRNA-seq of a *Streptococcus mutans* lab culture with a defined whole genome sequence (Fig. 1C).

From the genomic tRNA database (17), we found that almost all tRNA^Leu^(AAG) gene in bacteria occurs among the class of Bacilli (Fig. S1C and D). Within this class, all families among the order of Bacillales have *tRNA^Leu^(GAG)* gene exclusively, and several families in Lactobacillales have *tRNA^Leu^(AAG)* gene exclusively (Figs. 1D and S1E). At the RNA level, tRNA^Leu^(GAG) can read the synonymous Leu codons of CUU and CUC, but tRNA^Leu^(AAG) may only read CUU. Converting tRNA^Leu^(AAG) to tRNA^Leu^(IAG) allows it to read both CUU and CUC, and also CUA (18, 19). However, the CUA decoding capability of tRNA^Leu^(IAG) is not required for these bacteria because they also contain tRNA^Leu^(UAG) for reading CUA (Fig. 1E). As a parallel, I34 modification is widespread in eukaryotes among tRNAs for Leu, Thr, Val, Ala, Pro, Ser, Arg, Ile, Gly, yet is not required to read their synonymous A-ending codons, as another U34 tRNA is present in each of these amino acid groups that can read the respective A-ending codons (6, 20). Thus, these bacterial families represent a natural experiment to dissect the influence of inosine in genome evolution that is distinct from the original wobble hypothesis (21).

We compared the synonymous Leu codon usage between the families containing the *tRNA^Leu^(AAG)* or *tRNA^Leu^(GAG)* gene within the same order. Carnobacteriaceae, Enterococcaceae, and Lactobacillaceae are commensal bacteria in the intestine, while Streptococcaceae are commensal bacteria in the oral microbiome. All these genomes have high A/T content at the same level (Fig. S1F), consistent with the Leu-UUA being the most abundant Leu codon (Fig. 1F). An obvious difference in decoding capabilities of tRNA^Leu^(GAG) and tRNA^Leu^(IAG) is the expanded recognition of codon Leu-CUA for tRNA^Leu^(IAG). To our surprise, CUA usage is identical between *tRNA^Leu^(AAG)*-gene and *tRNA^Leu^(GAG)*-gene groups. Instead, usage of Leu-CUU and Leu-CUC is markedly increased in the *tRNA^Leu^(AAG)*-gene groups, at the expense of Leu-UUA, even though tRNA^Leu^(IAG) does not read UUA (Fig. 1G).

### tRNA^Leu^(IAG) equalizes decoding speed of CUU and CUC codons

**Figure 2:**
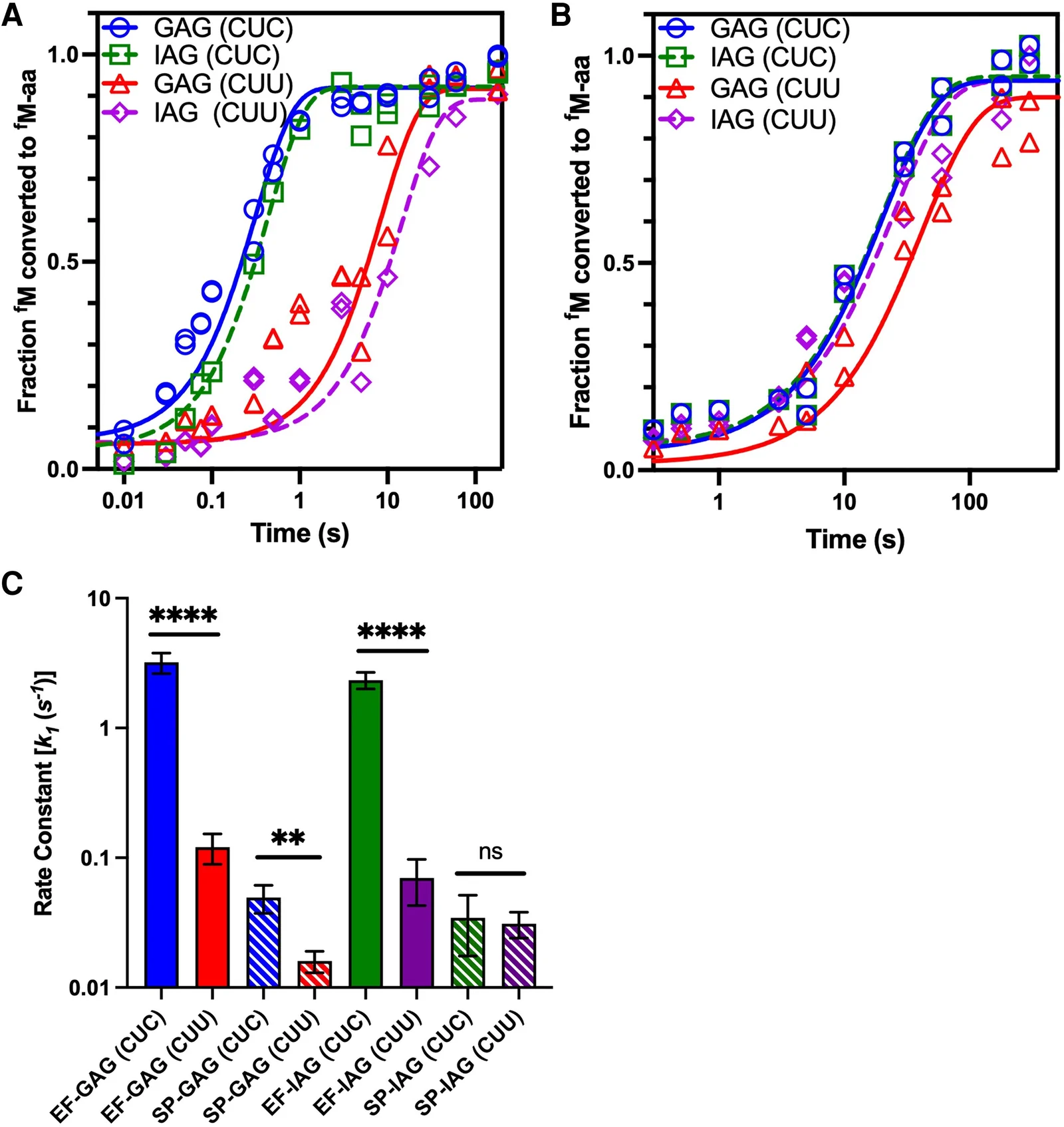
Inosine modification on SP tRNA^Leu^ equalizes decoding speed of CUU and CUC codons. In vitro translation assays were used to measure the rate of addition of leucine on CUU and CUC codons. 70S initiation complexes were reacted with either tRNA^Leu^(IAG) or tRNA^Leu^(GAG) derived from A) EF and B) SP. C) Rate constants for Met-Leu dipeptide formation for each combination of tRNA and substrate were calculated and compared. Rate constants and error bars derived from global fit of all time points (≥28 per tRNA:codon pair). Two sample, unpaired t test was performed for the tRNA from each species for the two codons (*n* ≥ 3) (*P*-values > 0.05 = ns, *P* ≤ 0.01 = **, *P* ≤ 0.00001 = ****).

tRNA modifications can affect both translation speed and fidelity (22, 23). Achieving the maximum translation speed at specific codons may not be the most advantageous, rather, the translation machinery aims to balance decoding speed at most codons to minimize ribosome collision and other events that trigger stress (24–26). We hypothesize that tRNA^Leu^(IAG) could affect the decoding speed of CUU versus CUC differently from tRNA^Leu^(GAG). Our hypothesis is based on a similar case where modulation of the tRNA queuosine modification affects synonymous codon choices in a eukaryotic genus (7), as well as a broad trend among eukaryotes to favor I34-modified tRNAs (6). We assessed the speed of amino acid addition by tRNAs from *Streptococcus pyogenes* (SP) representing tRNA^Leu^(IAG) and *Enterococcus faecalis* (EF) representing tRNA^Leu^(GAG) on CUU and CUC codons. To accomplish this, we used a well-established in vitro translation system that allows us to measure the formation of Met-Leu dipeptide over time using in vitro transcripts of tRNA^Leu^(GAG) and tRNA^Leu^(IAG) containing a single I34 modification (Fig. S2A and B) (27, 28). We observed that EF-tRNA^Leu^(GAG) adds leucine ∼10-fold faster on CUC than CUU (Fig. 2A). By contrast, SP-tRNA^Leu^(IAG) shows no preference on CUC relative to CUU (Fig. 2B). Introducing I34 into EF-tRNA did not affect relative amino acid addition speeds of CUU over CUC, whereas introducing G34 into SP-tRNA restored a ∼3-fold advantage to CUC over CUU (Fig. 2C).

Together, these results show that CUU and CUC are decoded at similar speed by the *Streptococcus* tRNA^Leu^(IAG), in contrast to the *Enterococcus* tRNA^Leu^(GAG) that decodes CUC much faster over CUU. For SP-tRNA^Leu^(IAG), inosine is necessary to equalize the decoding speed of CUU and CUC, while for EF-tRNA^Leu^(GAG), introducing I34 does not change the preference of CUC over CUU. This differential inosine effect on CUU/CUC decoding in these tRNAs may be attributed to the large sequence differences between SP-tRNA^Leu^(IAG) and EF-tRNA^Leu^(GAG) (Fig. S2B).

### tRNA^Leu^(IAG) confers a growth advantage

**Figure 3:**
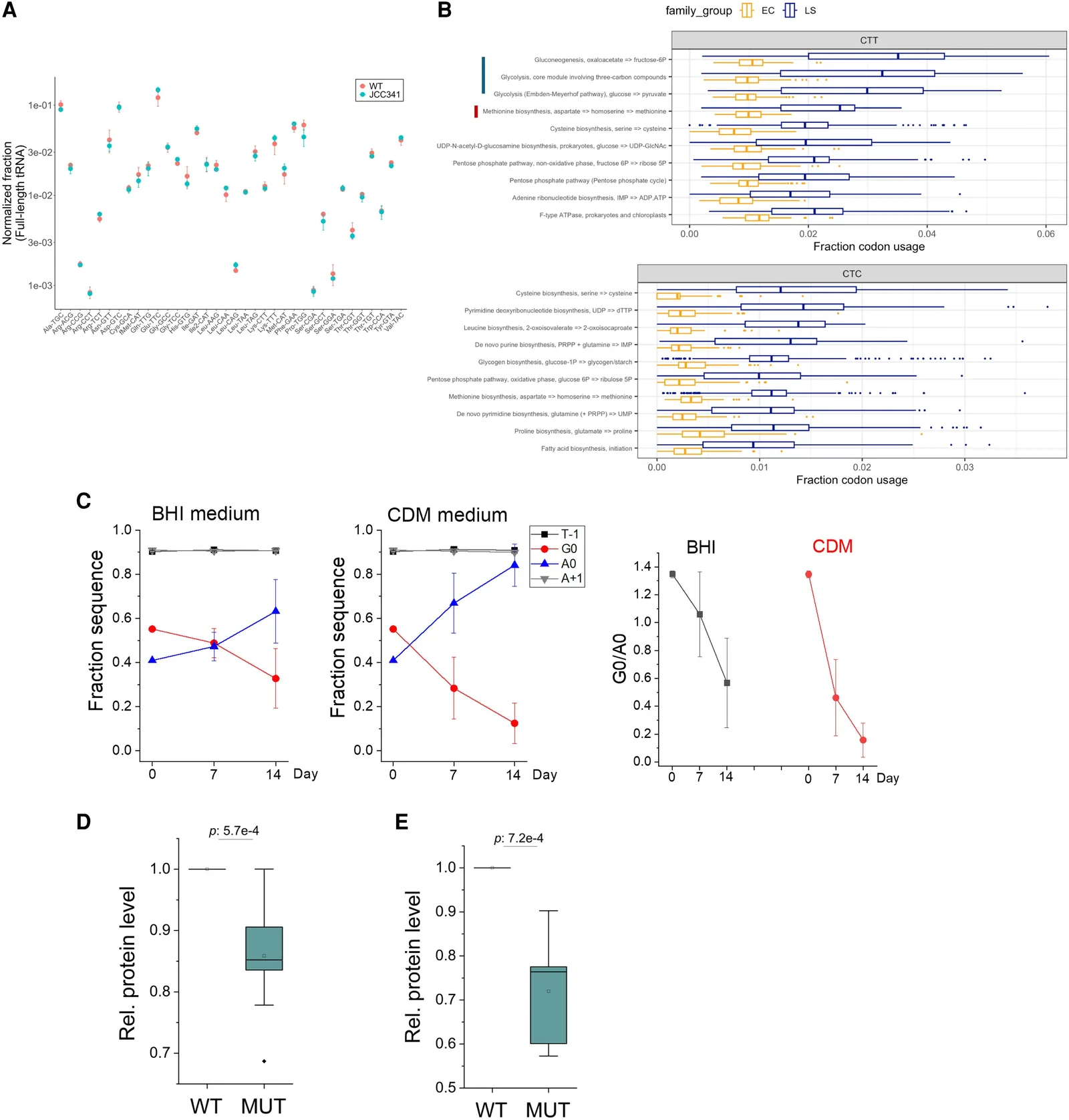
*Streptococcus* with tRNA^Leu^(AAG) outperforms its isogenic tRNA^Leu^(GAG) strain in growth. A) tRNA-seq shows identical expression of all tRNAs between the wild-type (WT) strain containing a *tRNA^Leu^(AAG)* gene and its isogeneic *tRNA^Leu^(GAG)* strain. B) Top 20 per-module relative frequencies of CUU and CUC relative to all Leucine codons. The modules are named with their KEGG accession. Box shows the three glycolysis modules. C) Growth competition of the WT-AAG and its isogenic mut-GAG strain. The mixed cultures started with 45% WT and 55% mutant at day 0. Total genomic DNA was extracted at days 7 and 14 for Saenger sequencing that identifies the proportion of the WT-AAG- and mut-GAG in the same mixture. BHI medium contains 0.2% glucose and CDM medium contains 1% glucose. D) Relative levels of the glycolysis enzymes (KEGG Module M00001, *n* = 10) in the WT and mutant strains (*n* = 3 biological replicates each) measured by proteomic mass spectrometry. E) Relative levels of the methionine biosynthesis enzymes (KEGG Module M00017, *n* = 7) in the WT and mutant strains (*n* = 3 biological replicates each) measured by proteomic mass spectrometry.

*Streptococcus mutans* is a commensal human bacterium commonly found in the oral cavity. *Streptococcus mutans* and other Streptococcus species metabolize sucrose to lactic acid, leading to decay of tooth enamel (29). We constructed a *S. mutans* strain containing a single mutation converting the native *tRNA^Leu^(AAG)* gene into *tRNA^Leu^(GAG)* in the whole genome (Fig. S3A). In standard culture media, this strain is viable with the same tRNA expression levels and growth dynamics (Fig. 3A), indicating that the expanded decoding potential of tRNA^Leu^(IAG) is not essential, although we cannot rule out other pleotropic effects upon mutating this tRNA.

Phenotypic consequences of tRNA decoding speed are often difficult to measure, as the effects are small, but accumulate over the course of translating many codons (30, 31). We asked which metabolic pathways show the largest difference in codon bias between *tRNA^Leu^(GAG)* and *tRNA^Leu^(AAG)*-containing bacterial families. The top three hits were all related to glucose metabolism (Figs. 3B and S3B). However, all 6 enzymes in KEGG module M00002 and 5 of the 7 enzymes in module M00003 are part of the 10 enzymes in module M00001, so all 3 modules can be represented by module M00001. Glycolytic enzymes are generally from high expression genes where selective pressure is strong and translation speed can have substantive fitness effects (30, 32–34). *Streptococcus mutans* particularly relies on glycolysis for energy production (29, 35). We therefore hypothesize that *S. mutans* strain containing the *tRNA^Leu^(GAG)* gene may produce glycolytic enzymes less efficiently compared to the wild-type strain containing the *tRNA^Leu^(AAG)* gene due to altered decoding of the abundant CUU codons in the glycolysis genes (Fig. 3B). To test this, we grew mixed cultures of WT and mutant strains in BHI medium containing 0.2% glucose and in CDM medium containing 1% glucose for up to 14 days. We collected the culture at days 0, 7, and 14 days, extracted total genomic DNA and performed Saenger sequencing to compare the WT and mutant strain populations (Figs. 3C and S3C). Indeed, the WT strain outperformed the mutant strain in both media, increasing its proportion from 45 to 67% from day 0 to day 14 in BHI, and from 45 to 88% from day 0 to day 14 in CDM that contained a higher level of glucose than BHI to support growth. These data support a phenotypic role for tRNA^Leu^(IAG) modification in decoding leucine codons in vivo, which likely facilitated genome-wide recoding of Leu codons in evolution.

tRNA wobble modification effects on synonymous codon changes can be examined at the translation level by ribosome profiling (36). However, it is more difficult to dissect synonymous codon effects in bacteria (37), especially for Streptococcus, a Gram-positive bacterium (38). Instead, we performed mass spectrometry proteomics to examine the differential effect of the wild-type and mutant strains at the proteome level under shifting glucose conditions. Grouping all 10 glycolysis enzymes in the KEGG module M00001 (representing the top three modules in Fig. 3B) identified a significantly lower level of protein expression of this group in the mutant strain with an average reduction of 15% (Fig. 3D). Nine of the 10 individual enzymes in this group showed reduced levels between 10 and 30% in the mutant strain (Fig. S3D). Similarly, grouping all seven methionine biosynthesis enzymes (representing the fourth module in Fig. 3B) showed a significant reduction of this group in the mutant strain at an average of 23% (Fig. 3E) and between 10 and 45% among the individual enzymes (Fig. S3E). These results demonstrate that the tRNA^Leu^(AAG) mutation indeed affects the protein levels in these pathways that are enriched in Leu CUU codons.

### tRNA^Leu^(IAG) facilitates UUA recoding

**Figure 4:**
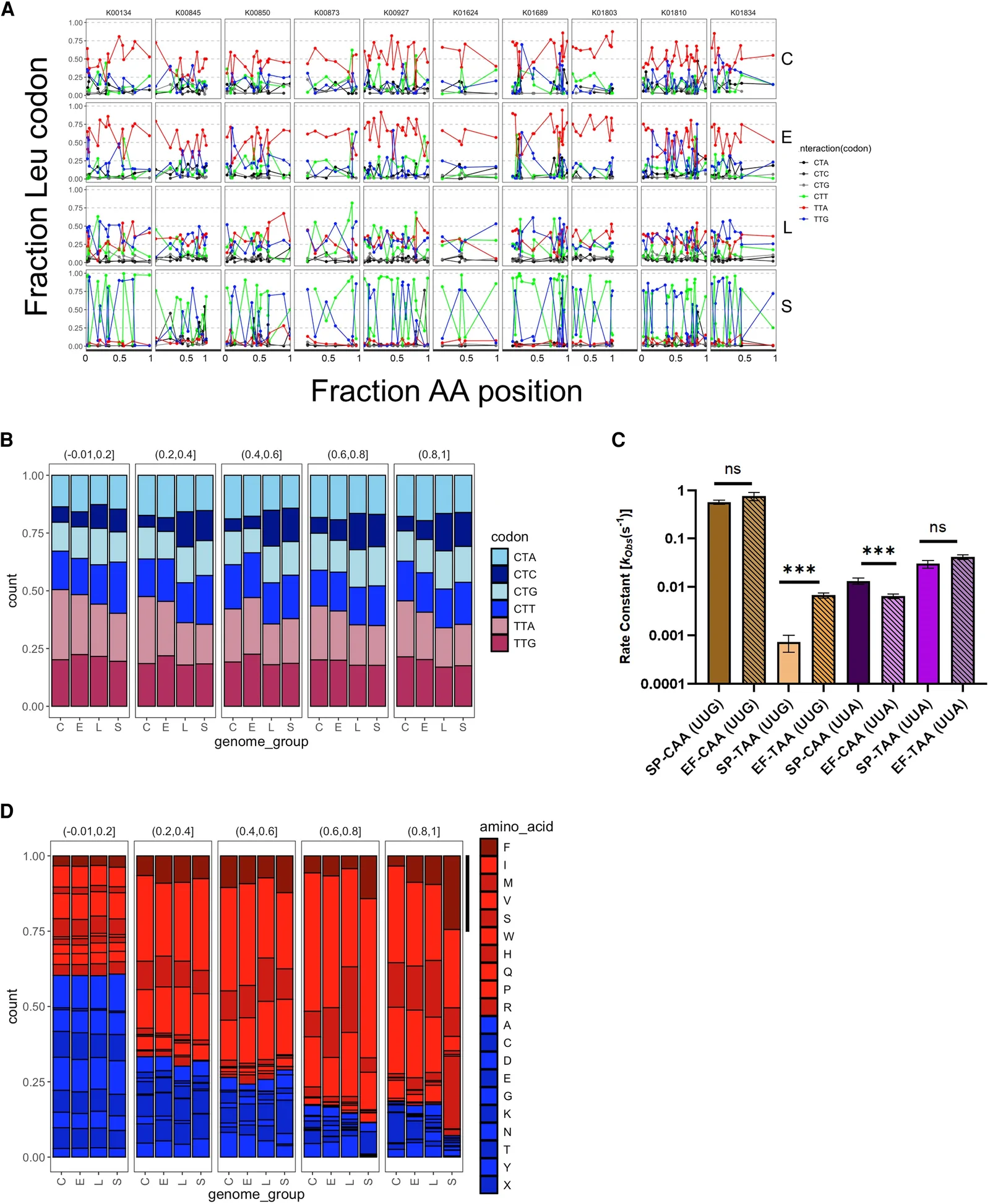
Slow decoding of UUA facilitates genome recoding. A) Synonymous Leu recoding of conserved sites in all 10 enzymes in glycolysis. Positions with >50% Leu conservation and >20 genomes are aligned. C, Carnobacteriaceae; E, Enterococcaceae; L, Lactobacillaceae; S, Streptococcaceae. B) Conservation of leucine versus codon bias among glycolytic genes. The labels at the top of the columns (eg (−0.01,0.2), (0.2,0.4]) refer to the “degree of Leu conservation” bins. C) Dipeptide formation rate constants with tRNA^Leu^(CAA) and tRNA^Leu^(UAA) derived from sequences of EF and *Streptococcus pyrogens* (SP). Dipeptide formation against two mRNA substrates containing coding sequence of Met-Leu(UUG)-Stop and Met-Leu(UUA)-Stop. Two sample, unpaired t test was performed for the tRNAs from the two species for each codon. *n* = 2–4 (*P* > 0.05; ns, *P* ≤ 0.05; *, *P* ≤ 0.01; **, *P* ≤ 0.001; ***, *P* ≤ 0.00001; ****). D) Nonsynonymous amino acid changes at all levels of Leucine conservation between two families with *tRNA^Leu^(AAG)* gene (L, S) and two with *tRNA^Leu^(GAG)* gene (C and E). Red shows nonsynonymous substitutions possible from a single mutation from Leu codons. Blue shows nonsynonymous substitutions requiring two or more mutations from Leu codons. Vertical bar upper right highlights the Phe codons.

We carried out additional analysis on the usage of Leu codons in the glycolysis pathway among the *tRNA^Leu^(AAG)*-and *tRNA^Leu^(GAG)* gene families. Synonymous codon usage is zero-sum, so increased Leu codon of CUU/CUC usage must draw from other Leu codons. All families in our analysis have equal levels of GC-content (Fig. S1F). The high AT-content of these genomes makes it likely that the most abundant and A/T-rich Leu codon, UUA, are replaced by increased CUU and CUC usages. We examined codon usage at sites where Leucine is conserved among the set of glycolytic enzymes in KEGG pathway M0001 and confirm that the same sites have indeed undergone synonymous recoding (Fig. 4A). Expanding upon this analysis, we grouped positions based on Leucine conservation and compared relative synonymous codon usage (RSCU) at each position. *Streptococcus* and *Lactobaccillaceae* family genomes show reduced usage of UUA codon at nearly every grade level of Leu conservation (Fig. 4B). This sort of codon bias, even in sites where Leucine identity is not strictly important, suggests selection for translation speed (20, 39).

What other selective pressure could drive synonymous UUA recoding? UUA is two mutations away from CUU/CUC (Fig. S4A). To get there one mutation at a time, UUA can be first mutated to UUG which can become terminal. Indeed, UUG is also over-represented in the L/S genomes at the same position as UUA in the C/E genomes (Fig. 4A). UUG in all these genomes can be read by another tRNA^Leu^(CAA). To test the speed of amino acid addition on UUA and UUG codons by *S. pyogenes* and *E. faecalis* tRNA^Leu^(UAA) and tRNA^Leu^(CAA), we again turned to our reconstituted translation assay. We find that SP-tRNA^Leu^(UAA) adds Leucine on UUA ∼2-fold slower than EF-tRNA^Leu^(UAA) (Fig. 4C). Both SP and EF-tRNA^Leu^(UAA) can still decode UUG according to the wobble rule in principle, but they add Leucine ∼50-fold slower on UUG compared to UUA. Furthermore, SP-tRNA^Leu^(UAA) adds Leucine on UUG ∼10-fold slower than EF-tRNA^Leu^(UAA) (Fig. 4C). By contrast, SP and EF-tRNA^Leu^(CAA) have similar activities when decoding UUG. These tRNA sequences are nearly identical, except for sequence and size differences of the variable loop (Fig. S4B), a tRNA region known to alter their activity in translation (40). In addition to reduced activity, we observe lower expression of tRNA^Leu^(UAA) among tRNA^Leu^ from the *Streptococcus* family species than *Enterococcus* in our oral microbiome samples (Fig. S4C). The relative tRNA^Leu^ expression level in the oral microbiome is also recapitulated in our *S. mutans* cultures (Fig. S4D). Both the reduced activity and a relatively lower expression level of tRNA^Leu^(UAA) in *Streptococcus* suggest substantially reduced decoding speed for UUA, consistent with a potential benefit for synonymous recoding of UUA to UUG in *Streptococcus*.

In the complex interplay of speed and accuracy, would mistranslation leave in the genome evolution in the context of *tRNA^Leu^(AAG)* gene appearance? We reasoned that in *Streptococcus* the UUA codon is more likely to be misdecoded by the near-cognate tRNA^Phe^(GAA) which only requires G:A pairing at the third codon position (41). Some sites may be adapted to be robust to this translational error—producing a functional protein with both a cognate Leu, or a mistranslated Phe. These sites would also be robust to transversion mutations over evolutionary time, showing an overabundance of Phe compared to other drift mutations at conserved Leu sites. We compared sites with different levels of Leucine conservation and selected genomes where the position is not Leucine. Amino acids that are a single mutation away from a Leu codon dominate at sites with >20% Leu conservation (Fig. 4D). When comparing bacterial families, *Streptococcus* shows over-representation of Leu-to-Phe at many conservation levels, consistent with robustness against near-cognate misdecoding (Fig. 4D), although alternative explanations, such as underlying local mutational biases could also explain this observation.

## Concluding remarks

Altogether, our results suggest a model of coding evolution by the appearance of tRNA^Leu^(IAG). UAA is a nonoptimal codon in *Streptococcus*—both due to slow decoding by its cognate tRNA or high risk of mistranslation by near-cognate tRNA. Both accuracy and speed of translation have been shown to be strong determinants of codon bias (33, 39). Pressure against UUA is relieved by the appearance of the *tRNA^Leu^(AAG)* gene where A34 can be readily modified to I34, as the A-to-I deaminase already exists in the same genome to generate tRNA^Arg^(ICG). The introduction of inosine modification increases the pool of tRNA^Leu^ available to decode CUA and facilitates genetic drift from UUA to CUA, which otherwise could not be supported by the tRNA^Leu^(UAG) alone. Once mutated to CUA, the equal decoding ability of tRNA^Leu^(IAG) can further facilitate codon drift to CUU or CUC for these codons to become fixed in the genome.

Does tRNA^Leu^-inosine-modification driven synonymous recoding in bacteria go beyond Lactobacillales (Phylum: Firmicutes)? A very brief survey of the tRNA^Leu^ genes in the genomic tRNA database (Fig. S1D) shows the presence of the tRNA^Leu^(AAG) gene in Mycoplasma (Phylum: Mycoplasmatota) and Prochlorococcus (Phylum: Cyanobacteria), suggesting that similar leucine codon recoding may be widespread in the bacterial kingdom.

## Materials and methods

### Experimental

#### Oral microbiome RNA extraction and sequencing

##### Ethical considerations

The study was approved by the institutional review board committee of the University of Chicago, Chicago, IL, USA [IRB19-0065; approved 2019 April 4]. Written informed consent was obtained from all individuals involved in this analysis.

##### Oral cavity

Tongue dorsum scrapings were collected from one female and three male volunteers (two samples per volunteer) on two consecutive days [A and B sample]. Sample collection used BreathRx Gentle Tongue Scraper (Philips Sonicare) and was performed in the morning hours prior to eating, drinking or performing oral hygiene. Starting as far back as possible on the tongue, the scraper was passed forward over the entire surface three sequential times. The scrapings were combined with 500-µL RNAlater Stabilization solution (Invitrogen, AM7020) and stored at −80 °C until extraction.

##### Total RNA extraction

RNAlater was first removed from tongue dorsum samples by centrifugation at 17,200 rcf for 10 min at 4 °C. Pelleted material was lysed in 400 μL of 0.3 M NaOAc/HOAc,10 mM EDTA, pH 4.8 with an equal volume of acetate-saturated phenol:chloroform pH 4.5 (Invitrogen, AM9722). After addition of 1.0 mm glass lysing beads (Bio-Spec Products, 11079110) in a 1:1 ratio (bead:sample weight), samples were placed in a reciprocating bead beater (Mini-Beadbeater-16, Bio-Spec Products) for two 1-min intervals on maximum intensity. Samples were centrifuged at 17,200 rcf for 15 min at 4 °C before re-extraction and isopropanol precipitation of total RNA. Pellets were washed with 75% ethanol before resuspension in an acid-buffered elution buffer (10 mM NaOAc, 1 mM EDTA, pH 4.8).

##### Microbiome tRNA sequencing

These were performed using MSR-seq method (13, 42) and total RNA above as inputs.

##### Microbiome tRNA analysis

The methods of the previously published pipeline (1) were used with some modifications. Raw paired-end sequence reads of 75 or 100 nucleotides were processed by Illumina-utils (43). Inserts contained a seven nucleotide sample barcode and a random six nucleotide unique molecular identifier (UMI). Given that tRNA molecules range in length from 74 to 96 nucleotides, forward and reverse 100 nucleotide reads fully covered some tRNA sequences and partially overlapped for others. We upgraded the Illumina-utils “iu-merge-pairs” command to merge both fully and partially overlapping reads, while trimming overhanging adapter sequences in the case of more than full overlap (the command line flag, “–marker-gene-stringent, “ enables consideration of full as well as partial overlap). We minimized erroneous base calls, important for our analysis of modification-induced mutations, by retaining reads that matched with zero mismatches in the overlapping region (option “–max-num-mismatches 0') (43).

The procedure for de novo profiling of full-length and fragmentary tRNA reads introduced in tRNA-seq-tools (1) was modified as follows. tRNA structural features were identified from the 3′ end of the read. The minimum criteria for tRNA identification were the correct distance to the 7 canonically conserved nucleotides in the T arm, of which 5 must be found, and base pairing in of the T stem. A full-length read must contain a base-paired acceptor stem and all features in between.

tRNA sequences were taxonomically annotated by using the Global Alignment for Sequence Taxonomy tool (44) to search a set of reference tRNA sequences that tRNAscan-SE (v1.3.1) (45) identified from 4,235 gold-standard bacterial genomes (nonendosymbiont genomes with an assembly level of “chromosome”) stored in the Ensembl Genomes 2016 database (46).

#### Recombinant Streptococcus TadA deaminase purification and in vitro assay

SpTadA was overexpressed in BL21(DE3) and purified by immobilized metal affinity chromatography to a final concentration of 245 μM (5.2 mg/mL). tRNA substrates were prepared by in vitro transcription. The assays were carried out in a deamination buffer (25 mM KCl, 2.5 mM MgCl_2_, 2 mM dithiothreitol, 50 mM Tris HCl, and 10% (v/v) glycerol; pH 7.5). To a total reaction volume of 20 μL, 170 nM tRNA (100 ng), 150 nM SpTadA, and 1 U RiboGuard RNase Inhibitor (Lucigen) were added and incubated at 37 °C for 3 h before quenched by heating up to 95 °C for 10 min. Control reactions were prepared by omitting the enzyme. tRNA was diluted, reverse transcribed, amplified by polymerase chain reaction, and analyzed by Sanger sequencing.

#### Reconstituted in vitro assay to measure the speed of amino acid addition

We used a well-established reconstituted in vitro assay to measure the rate of amino acid addition on leucine and codons (47). *Escherichia coli* ribosomes, initiation factors, elongation factors and tRNA^fMet^ were purified as previously described (47). 70S initiation complexes with ^35^S-^f^Met-tRNA^fMet^ positioned in the P site were assembled on PAGE-purified T7-transcribed mRNAs possessing 5′ and 3′ UTRs (27, 47). Equal volumes of ternary complex containing EF-Tu, 5 mM GTP and 6 mM aminoacylated tRNAs of interest were added to purified initiation complexes (200 nM) to begin amino acid addition. tRNAs were aminoacylated either by *E. coli* Leu-tRNA aminoacyl synthetase (LeuRS) in the presence of low levels of DMSO (<10%), or the flexizyme (48). Discrete time points (0–600 s) were quenched by 500 mM KOH (final concentration) either by hand or using a KinTek quench-flow apparatus. ^35^S-^f^Met reactants were separated from ^35^S-^f^Met-Leu products by electrophoretic TLC and visualized by phosphorimaging. Images were quantified with ImageQuant (GE). The fraction product formed as a function of time were fit using equation below to obtain the observed rate constants for amino acid addition (*k*_obs_):


$$\mathrm{Fraction}\;\mathrm{product}=A\times (1-e^{k_{\mathrm{obs}}\times t})$$

#### Mutation of tRNA^Leu^(AAG) gene and whole genome sequencing validation

To introduce the nucleotide mutation into the genome, ∼3 kb upstream and downstream of the tRNA^Leu^(AAG) gene (SMU_t19) were amplified using primer sets JC751/JC752 (Table S1) and JC753/JC754, respectively. These fragments were fused in a second PCR reaction, and the resulting ∼6 kb fragment containing the mutation was amplified using primers JC755/JC756. The linear DNA was used to transform wild-type UA159 according to the protocol of Junges et al. (49) using synthetic XIP (sequence GLDWWSL; NeoScientific). To identify the mutation in the resulting transformants, sense primers with the wild-type (JC757) or mutated (JC758) nucleotide at the 3′ terminus were selected and, together with a common antisense primer (JC759), PCR annealing conditions were optimized such that only correct base pairing at the 3′ position would produce product. Transformants were initially screened by colony PCR, then genomic DNA was prepared from positive clones (MasterPure Gram Positive DNA Purification Kit; Biosearch Technologies), and the mutation was confirmed by Sanger sequencing of the genomic region.

#### *Streptococcus mutans* experiments

##### Culture and competition

*Streptococcus mutans* strains were routinely cultured in Todd Hewitt Broth (RPI International) + 0.2% yeast extract (VWR) at 37 °C with 5% CO_2_. For transformations, strains were grown in a Chemically Defined Medium (CDM) (50) supplemented with 1% w/v glucose. For storage at −80 °C, sterile glycerol was added to a final concentration of 20%.

For the competition experiment, wild-type UA159 and its isogenic tRNA mutant (JCC341) were picked from brain heart infusion (BHI; Difco) agar plates and grown overnight in BHI at 37 °C + 5% CO_2_. The next day, the cultures were mixed 1:1 (using three independent overnight cultures of each strain) and an aliquot of each culture was saved for genomic DNA isolation. The mixed cultures were then diluted to an OD600 of 0.01 in BHI (peptide-rich, 0.2% glucose) or CDM (peptide-free, 1% glucose) and incubated at 37 °C + 5% CO_2_. Every 24 h, an aliquot of each competition culture was removed for genomic DNA isolation, and the cultures were back diluted to OD_600_ = 0.01 in their respective media; serial passaging was repeated for 14 days. To assess the amount of wild-type *S. mutans* versus tRNA mutant at each time point, genomic DNA was isolated, and a ∼3.4 kb fragment containing the tRNA gene was amplified by PCR using primers JC755/JC759 then subjected to Sanger sequencing with JC778. The relative amount of each population was calculated by the Saenger sequencing peak density for A, G, C, T at each position.

##### RNA extraction and tRNA sequencing

*Streptococcus mutans* starter cultures were prepared by growing strains of interest in Brain Heart Infusion Broth without Dextrose (BHI-D; Alpha Biosciences) to mid-log phase. The cells were concentrated by centrifugation, and the pellets were resuspended in BHI-D + 20% glycerol, and stored as single-use aliquots at −80 °C. To collect RNA, a single-use starter culture for each strain was thawed, resuspended in BHI-D, and used to inoculate BHI-D + 0.15% glucose before incubation at 37 °C. Cells were harvested by centrifugation at 1 h. The pellet was resuspended in RNAlater (Invitrogen), incubated overnight at 4 °C, then the cells were centrifuged again and stored at −20 °C until further processing. RNA was isolated using the Zymo Quick-RNA Fungal/Bacterial Microprep Kit, treated with DNAse I according to the DNAfree kit (Invitrogen), then stored at −80 °C.

tRNA sequencing was performed using MSR-seq method and analysis pipeline (13, 42) and total RNA above as inputs.

##### Proteomics

*Streptococcus mutans* starter cultures prepared in BHI-D as described above were thawed, resuspended in BHI-D, used to inoculate 10 mL of fresh BHI-D to an OD_600_ of ∼0.03 and incubated at 37 °C + 5% CO_2_. Once the cells had doubled, the culture volume was expanded to 90 ml in fresh, prewarmed BHI-D. When the OD_600_ reached ∼0.06, 5 mL of filter-sterilized BHI + 9.5% glucose was added to each culture to yield a final glucose concentration of 0.5%. After 30 min, the cells were harvested by centrifugation, washed once with ice cold PBS, then the pellets were flash-frozen and stored at −80 °C until mass spec analysis.

For proteomic MS, samples were lyzed with 6 M guanidine hydrochloride in 0.2 M Tris pH 8.0 using a tissue lyser and glass beads. The samples were then reduced and alkylated with TCEP and chloroacetaminde for 10 min at 95 °C. Proteins were precipitated overnight with 100% methanol at −20 °C. The pellets were digested in 50 mM EPPS with Lys-C for 2 h, then trypsin for overnight digestion at 37 °C. Each digest was run by nanoLC-MS/MS using a Vanquish Neo HPLC on a 50 cm μPAC + column feeding into an Thermo Orbitrap Astral mass spectrometer. The data were acquired using data-dependent acquisition.

The quantitation of the proteins was done using Proteome Discoverer (Thermofisher; version 3.2). All MS/MS samples were searched using Sequest set up to use cRAP database (to remove contaminants) and Uniprot-refprot_Streptococcus_mutans_UP000002512_20260123 (1953 entries) assuming the digestion enzyme trypsin. Sequest was searched with a fragment ion mass tolerance of 0.02 Da and a parent ion tolerance of 10.0 PPM. Carbamidomethyl of cysteine was specified as fixed modification. Sequest was searched including oxidation of methionine as variable modification followed by INFERYS rescoring. Deamidated of asparagine and glutamine, oxidation of methionine and N-term protein acetylation were specified as variable modifications in a second Sequest search. Peptides were validated by Percolator with a 0.01 posterior error probability threshold. The data were searched using a decoy database to set the false discovery rate to 1% (high confidence).

The peptides were quantified using the precursor abundance based on intensity. The peak abundance was normalized using total peptide amount. The peptide group abundances are summed for each sample and the maximum sum for all files is determined. The normalization factor used is the factor of the sum of the sample and the maximum sum in all files. Scaled abundances are also reported. The abundances are adjusted so that the average of the abundances is equal to 100 for each sample. Only proteins identified with at least two peptides and two peptide-spectral match and quantified in 50% of replicates were reported. Low resampling was used for imputation mode. The protein ratios are calculated using summed abundance for each replicate separately and the geometric median of the resulting ratios is used as the protein ratios. The significance of differential expression is tested using a t test with adjusted *P*-values based on the Benjamini–Hochberg method.

### Computational

#### Phylogenetic analysis

We utilized the anvi’o contigs databases for the Genome Taxonomy Database (GTDB) v220 (51, 52) that were included in the GlobDB v220 (53). We used anvi’o to perform a phylogenomic analysis of the 1,458 genomes in GTDB that resolved to families Aerococcaceae, Alicyclobacillaceae, Bacillaceae/Bacillaceae_G, Carnobacteriaceae, Enterococcaceae, Lactobacillaceae, Listeriaceae, Paenibacillaceae, Planococcaceae, Sporolactobacillaceae, Staphylococcaceae, and Streptococcaceae. For this analysis, we first used the program “anvi-get-sequences-for-hmm-hits” (54) to recover amino acid sequences for genes that matched to 18 ribosomal proteins that were annotated in at least 95% of the 1,458 genomes (Ribosomal_L1, Ribosomal_L13, Ribosomal_L17, Ribosomal_L19, Ribosomal_L20, Ribosomal_L21p, Ribosomal_L27, Ribosomal_L27A, Ribosomal_L9_C, Ribosomal_S10, Ribosomal_S11, Ribosomal_S13, Ribosomal_S15, Ribosomal_S16, Ribosomal_S2, Ribosomal_S20p, Ribosomal_S6, and Ribosomal_S9). We used “anvi-get-sequences-for-hmm-hits” with the parameters (i) “–max-num-genes-missing-from-bin 3′, which eliminated 103 low-quality genomes that were missing more than 3 of the 18 ribosomal proteins,” (ii) “–return-best-hit,” to ensure only one sequence per ribosomal protein per genome, and (iii) “–concatenate-genes” so the program used MUSCLE (55) to individually align each set of sequences that belonged to the same ribosomal protein gene family prior to reporting a FASTA file of concatenated alignments for downstream analyses. We then trimmed the resulting FASTA file using trimAl (56) and used IQTree 2 (57) with the WAG model and 1,000 ultrafast bootstraps (58) to compute the phylogenomic tree. We visualized the tree using the anvi’o program “anvi-interactive” in “—manual” mode and refined the figures in Inkscape (https://inkscape.org/).

#### Microbial tRNA gene and codon usage analysis

To investigate the prevalence of A34 in tRNA genes widely, the set of annotated tRNA genes associated with many sequenced organisms was downloaded from gtrnadb with the following command: wget http://gtrnadb.ucsc.edu/download/GtRNAdb/search/gtrnadb-search134145.fa.

This dataset was parsed using custom R script, and tRNAs an “A” in the first position of the annotated anticodon were selected and plotted. The set of Leu encoding A34 genes were then taken and the taxonomic genus annotated with each sequence was also plotted.

Oral microbiome reads were processed as above and mapped against a reference set of tRNAs for Streptococcus thermophilus (there is little sequence diversity among Leu tRNAs, so the choice of species/strain was not substantive). The mutation rate at genomic position A34 was plotted for all eight samples, revealing mutation to “G” nucleotide.

To investigate the codon usage among species in the order Lactobacillales, the genome-wide codon usage for NCBI-annotated organism was retrieved from CoCoPUTS (59). These data were combined with full taxonomic lineage from NCBI based on each unique taxon-id. Strains were combined into a single species representation by taking the median usage for each codon. Only species with more than 1,000 genes annotated were used. For each family in Lactobacillales, each species uses codons with some frequency. The species in a family thus represent distribution of codon usage for that family. These distributions were compared between families.

#### KEGG pathway codon analysis

We annotated each GTDB genome from the families Carnobacteriaceae, Enterococcaceae, Lactobacillaceae, and Streptococcaceae with KOfam functions using “anvi-run-kegg-kofams,” assigned their genes to metabolic pathways using “anvi-estimate-metabolism” (60), and counted the number of each codon per gene call using “anvi-get-codon-frequencies.” We examined codon frequencies at the genome and metabolic module levels.

We examined the codon frequencies per genome by doing the following, for each genome: (i) we kept only genes with length ≥900 bp (300 aa); (ii) for each nonstop codon, we summed the codon counts from all genes to get a per-genome codon count, and computed codon frequency relative to all codons or relative to Leucine codons as the per-genome codon count divided by the total count of all codons or all Leucine codons in the genome.

We examined codon frequencies at the level of individual metabolic module by implementing the following steps for each genome: (i) for each module we determined which gene calls belong to each module. This includes all genes with a KOfam annotation that is in the module definition, even if some of these genes have duplicate annotations or are enzymes that can catalyze the same reaction in the metabolic pathway. (ii) We summed up the codon counts of all genes in the module to get a per-module codon count. Here, we effectively treated the module as one long gene sequence. Therefore, we did not eliminate shorter genes in this analysis as we did above. (iii) We computed codon frequency relative to all codons as: the per-module codon count divided by the total count of all codons in the module. (iv) We computed codon frequency relative to Leucine codons as the per-module codon count divided by the total count of all Leucine codons in the module. We then extracted only the codon frequencies of CTC and CTT, eliminated any module that was present in <50% of all genomes, and computed the mean per-module codon frequency of each genome group, keeping only the modules with the top 20 largest mean difference between the “LS” and “EC” groups.

#### Leucine codon analysis in glycolysis

For the 1,458 genomes annotated for phylogenetic analysis, we further annotated protein coding regions and assigned them to 2683 KEGG Ontology pathways (61). Among all KO genes, DNA sequences are converted to protein sequences, then a multiple sequence alignment file is created for each gene ID using ClustalO, then amino acids are converted back to DNA to make a codon-aware alignment. For analysis, genes are limited to those in the KEGG glycolysis pathway, then only positions with >50% conservation of Leucine and >20 genomes from each family represented are considered. The rate of each Leu codon at each of these sites is plotted.

From the codon-aware multiple sequence alignment, only positions with >20 genomes from each family aligned were considered. Positions were grouped based on their degree of Leu conservation (0, 20, 40, 60, 80, and 100%). Then, the relative usage of each Leu codon was plotted for each family.

From the codon-aware multiple sequence alignment, only positions with >20 genomes from each family aligned were considered. Aligned positions were grouped based on their degree of Leu conservation as above. Then at each position, the rate of other, non-Leu, amino acid usage at that position was plotted.

## Supplementary Material

pgag187_Supplementary_Data

## Data Availability

The oral microbiome sequencing data and *S. mutans* culture MSR-seq data are deposited to NCBI GEO, accession GSE304916. Custom scripts for read processing are available at https://github.com/ckatanski/CHRIS-seq. Software related to taxonomic classification of tRNAs, genome- and metabolism-level characterization of codon frequencies, and phylogenomic analyses are available at https://github.com/merenlab/anvio/.
