# Supplementary material for: A transfer RNA inosine modification drives genome-wide synonymous recoding across human commensal bacterial families
Source: PNAS Nexus. 2026 May 30;5(6):pgag187. doi: 10.1093/pnasnexus/pgag187 (PMC13249217; doi:10.1093/pnasnexus/pgag187)
Supplement: pgag187_Supplementary_Data [file pgag187_supplementary_data.pptx]

## Slide 1
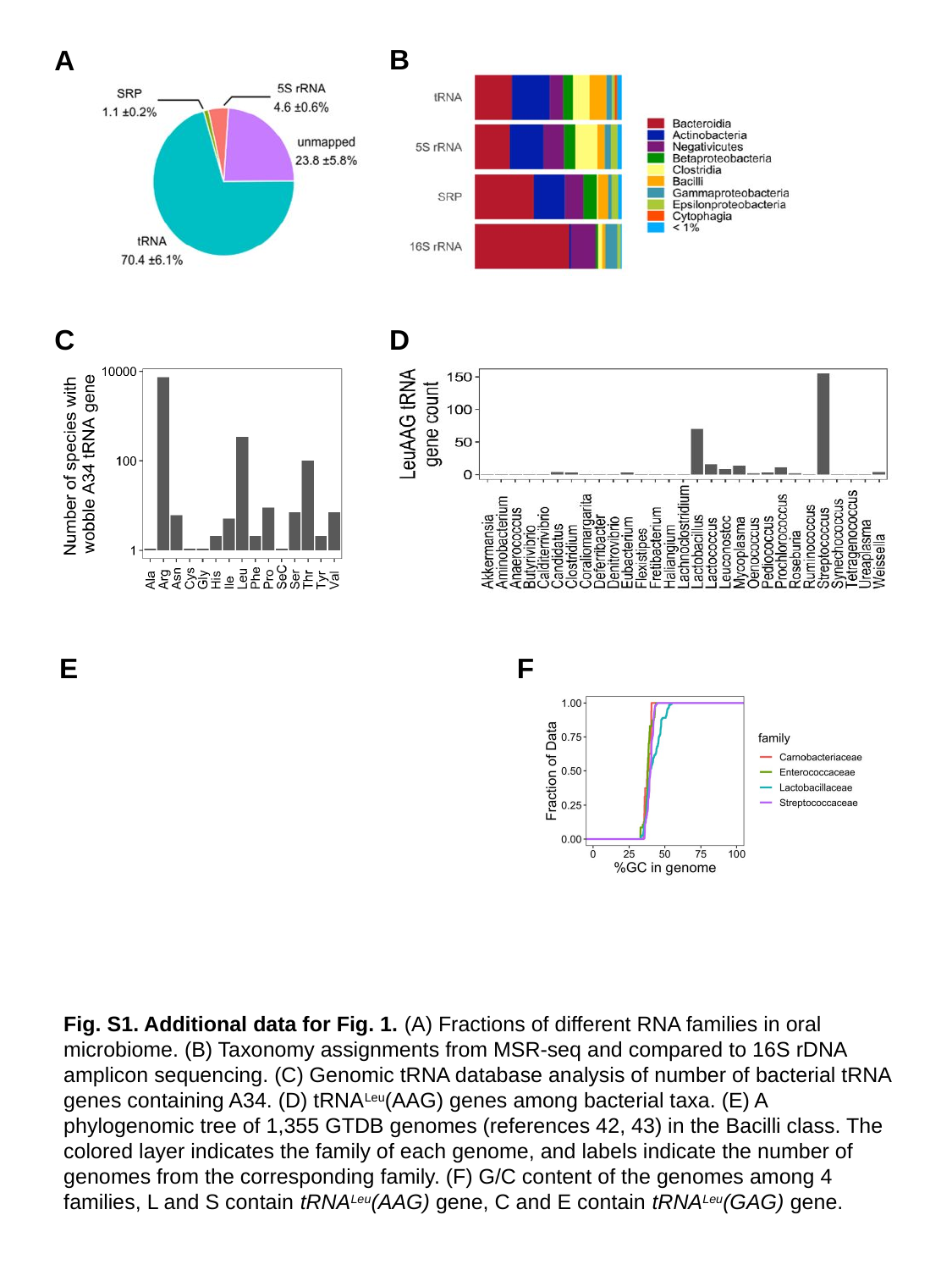

B
A
D
C
E
F
Fig. S1. Additional data for Fig. 1. (A) Fractions of different RNA families in oral microbiome. (B) Taxonomy assignments from MSR-seq and compared to 16S rDNA amplicon sequencing. (C) Genomic tRNA database analysis of number of bacterial tRNA genes containing A34. (D) tRNALeu(AAG) genes among bacterial taxa. (E) A phylogenomic tree of 1,355 GTDB genomes (references 42, 43) in the Bacilli class. The colored layer indicates the family of each genome, and labels indicate the number of genomes from the corresponding family. (F) G/C content of the genomes among 4 families, L and S contain tRNALeu(AAG) gene, C and E contain tRNALeu(GAG) gene.

## Slide 2
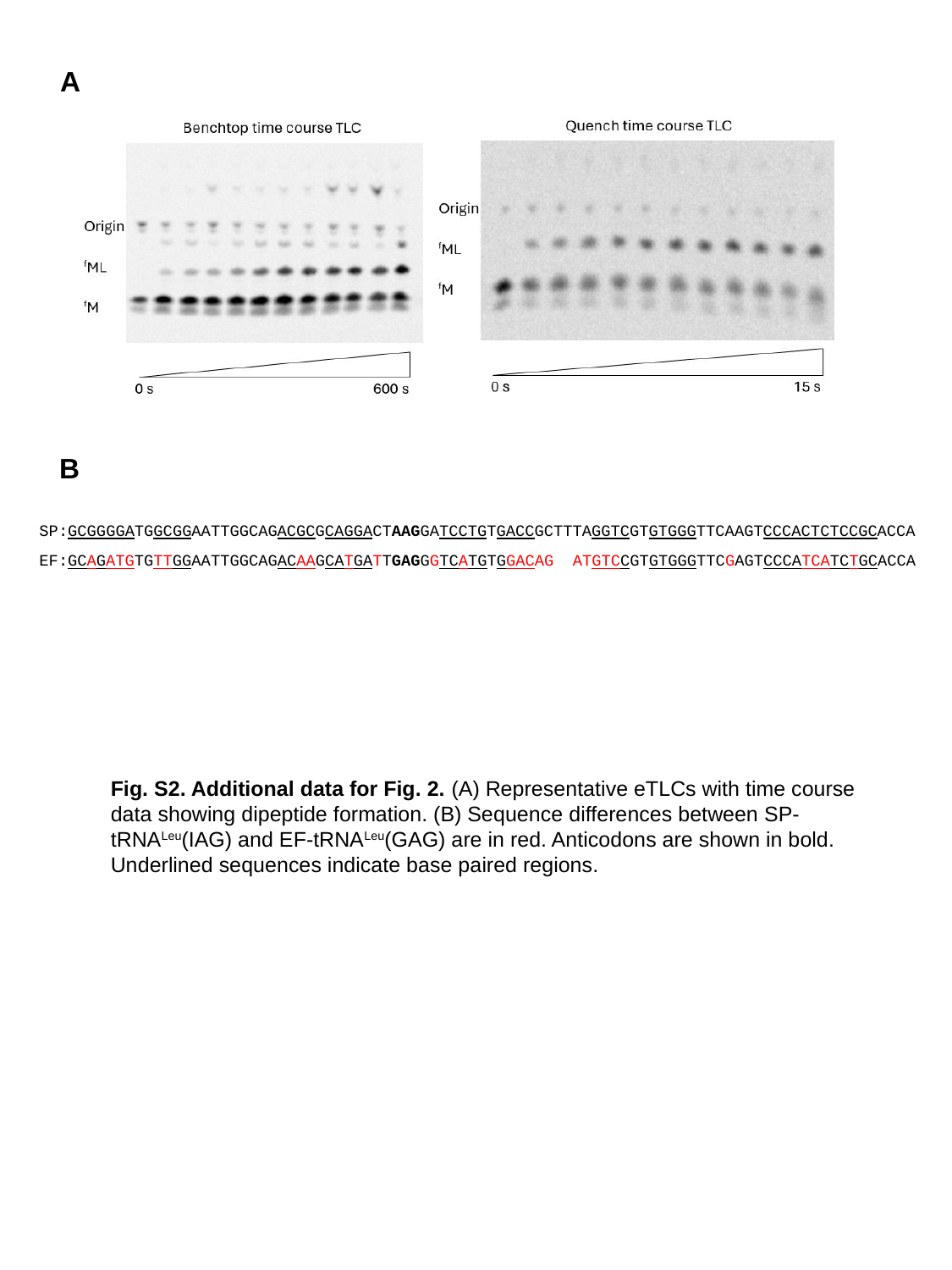

A
B
SP:GCGGGGATGGCGGAATTGGCAGACGCGCAGGACTAAGGATCCTGTGACCGCTTTAGGTCGTGTGGGTTCAAGTCCCACTCTCCGCACCA
EF:GCAGATGTGTTGGAATTGGCAGACAAGCATGATTGAGGGTCATGTGGACAG ATGTCCGTGTGGGTTCGAGTCCCATCATCTGCACCA
Fig. S2. Additional data for Fig. 2. (A) Representative eTLCs with time course data showing dipeptide formation. (B) Sequence differences between SP-tRNALeu(IAG) and EF-tRNALeu(GAG) are in red. Anticodons are shown in bold. Underlined sequences indicate base paired regions.

## Slide 3
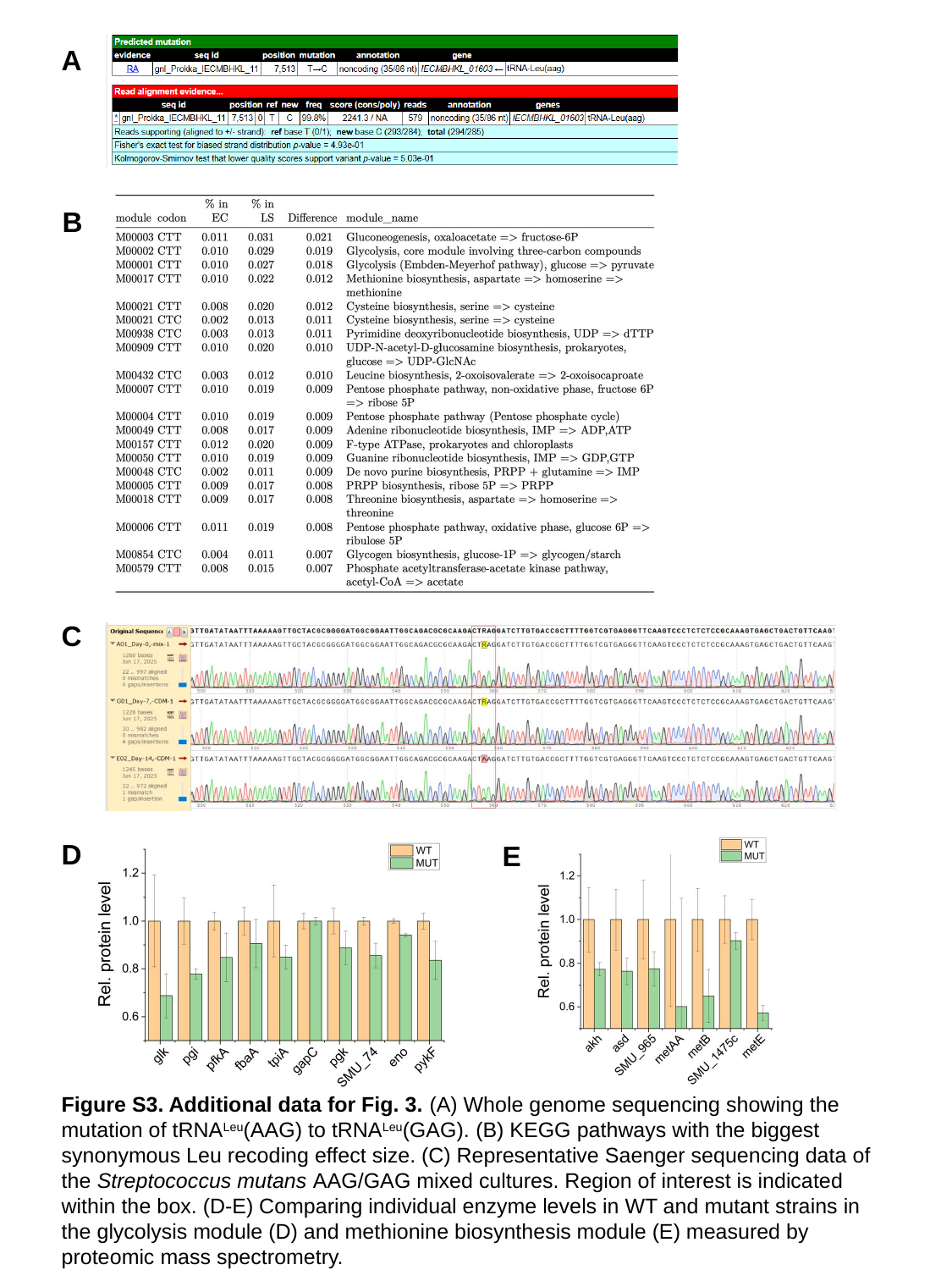

A
B
C
D
E
Figure S3. Additional data for Fig. 3. (A) Whole genome sequencing showing the mutation of tRNALeu(AAG) to tRNALeu(GAG). (B) KEGG pathways with the biggest synonymous Leu recoding effect size. (C) Representative Saenger sequencing data of the Streptococcus mutans AAG/GAG mixed cultures. Region of interest is indicated within the box. (D-E) Comparing individual enzyme levels in WT and mutant strains in the glycolysis module (D) and methionine biosynthesis module (E) measured by proteomic mass spectrometry.

## Slide 4
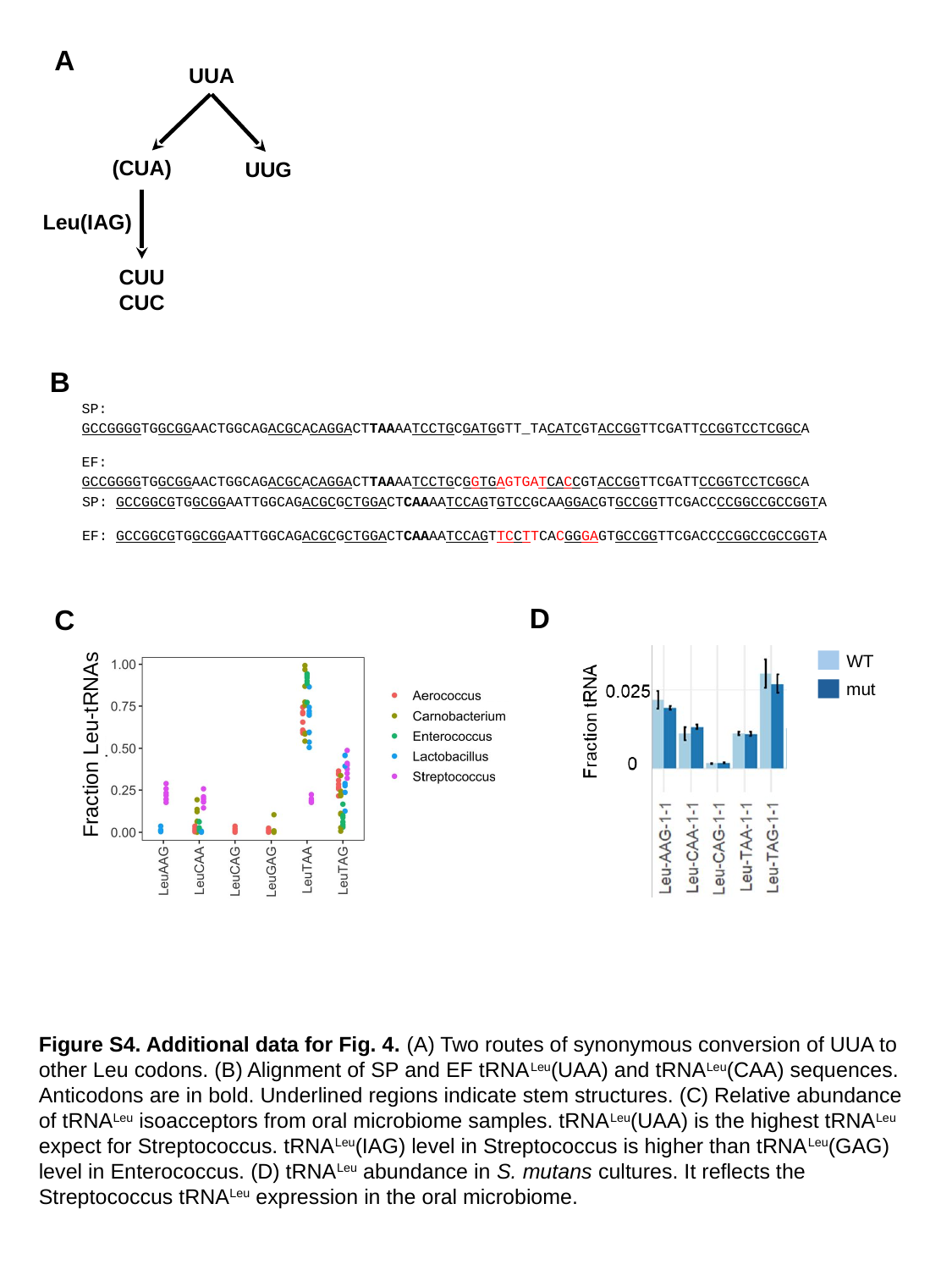

A
UUA
(CUA)
UUG
Leu(IAG)
CUU
CUC
B
SP: GCCGGGGTGGCGGAACTGGCAGACGCACAGGACTTAAAATCCTGCGATGGTT_TACATCGTACCGGTTCGATTCCGGTCCTCGGCA
EF: GCCGGGGTGGCGGAACTGGCAGACGCACAGGACTTAAAATCCTGCGGTGAGTGATCACCGTACCGGTTCGATTCCGGTCCTCGGCA
SP: GCCGGCGTGGCGGAATTGGCAGACGCGCTGGACTCAAAATCCAGTGTCCGCAAGGACGTGCCGGTTCGACCCCGGCCGCCGGTA
EF: GCCGGCGTGGCGGAATTGGCAGACGCGCTGGACTCAAAATCCAGTTCCTTCACGGGAGTGCCGGTTCGACCCCGGCCGCCGGTA
D
C
WT
mut
Fraction Leu-tRNAs
Figure S4. Additional data for Fig. 4. (A) Two routes of synonymous conversion of UUA to other Leu codons. (B) Alignment of SP and EF tRNALeu(UAA) and tRNALeu(CAA) sequences. Anticodons are in bold. Underlined regions indicate stem structures. (C) Relative abundance of tRNALeu isoacceptors from oral microbiome samples. tRNALeu(UAA) is the highest tRNALeu expect for Streptococcus. tRNALeu(IAG) level in Streptococcus is higher than tRNALeu(GAG) level in Enterococcus. (D) tRNALeu abundance in S. mutans cultures. It reflects the Streptococcus tRNALeu expression in the oral microbiome.

## Slide 5
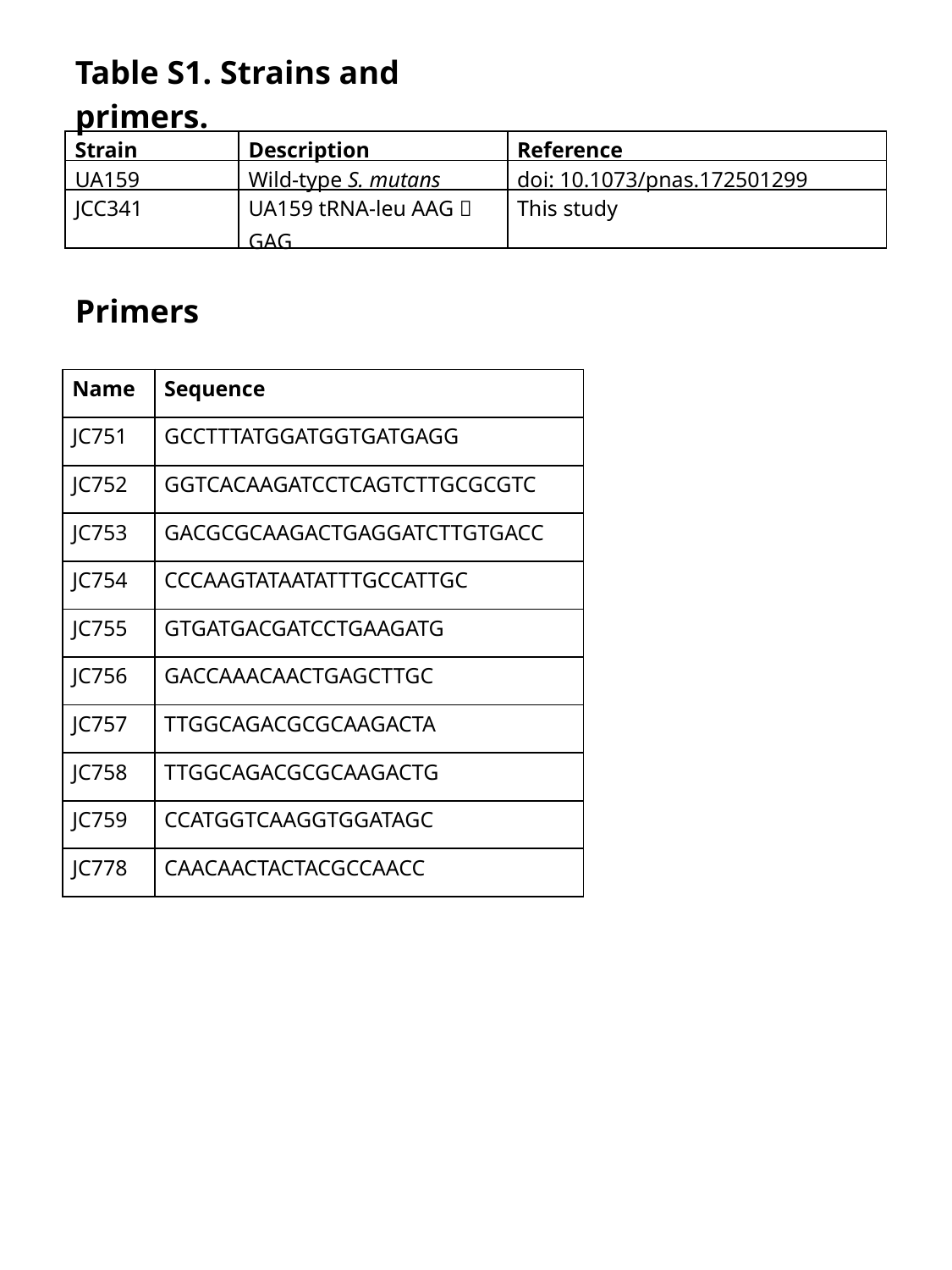

Table S1. Strains and primers.
| Strain | Description | Reference |
| --- | --- | --- |
| UA159 | Wild-type S. mutans | doi: 10.1073/pnas.172501299 |
| JCC341 | UA159 tRNA-leu AAG  GAG | This study |
Primers
| Name | Sequence |
| --- | --- |
| JC751 | GCCTTTATGGATGGTGATGAGG |
| JC752 | GGTCACAAGATCCTCAGTCTTGCGCGTC |
| JC753 | GACGCGCAAGACTGAGGATCTTGTGACC |
| JC754 | CCCAAGTATAATATTTGCCATTGC |
| JC755 | GTGATGACGATCCTGAAGATG |
| JC756 | GACCAAACAACTGAGCTTGC |
| JC757 | TTGGCAGACGCGCAAGACTA |
| JC758 | TTGGCAGACGCGCAAGACTG |
| JC759 | CCATGGTCAAGGTGGATAGC |
| JC778 | CAACAACTACTACGCCAACC |
